# Layered gadolinium hydroxides for low-temperature magnetic cooling†

**DOI:** 10.1039/c5cc05150a

**Published:** 2015-07-29

**Authors:** Gonzalo Abellán, Guillermo Mínguez Espallargas, Giulia Lorusso, Marco Evangelisti, Eugenio Coronado

**Affiliations:** a Instituto de Ciencia Molecular (ICMol), Universidad de Valencia 46980 Valencia Spain eugenio.coronado@uv.es; b Instituto de Ciencia de Materiales de Aragón (ICMA) and Departamento de Física de la Materia Condensada, CSIC–Universidad de Zaragoza Pedro Cerbuna 12 50009 Zaragoza Spain evange@unizar.es

## Abstract

Layered gadolinium hydroxides have revealed to be excellent candidates for cryogenic magnetic refrigeration. These materials behave as pure 2D magnetic systems with a Heisenberg–Ising critical crossover, induced by dipolar interactions. This 2D character and the possibility offered by these materials to be delaminated open the possibility of rapid heat dissipation upon substrate deposition.

## Abstract 2

A 2D gadolinium hydroxide with a large magnetocaloric effect is an excellent candidate for cryogenic magnetic refrigeration.

## Graphical abstract

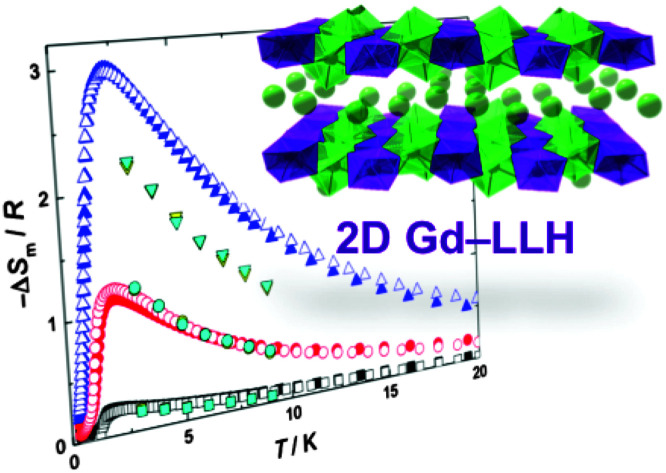

Two-dimensional (2D) materials have been attracting increasing interest in the last few years.^1^ Beyond graphene, layered metal hydroxides are promising candidates due to their chemical versatility and wide range of physical properties.^2–4^ These materials present host–guest anion exchange properties allowing the intercalation of stimuli-responsive molecules that can be used for controlling their physical properties.^4–6^ Moreover, they can be exfoliated into unilamellar nanosheets offering a plethora of different applications in sensors, energy storage and conversion, or magnetism, to name a few.^7^ One of the newest families of compounds that have emerged are layered lanthanide hydroxides (LLHs), which can be described by the general formula Ln_2_(OH)_5_A·*n*H_2_O, where A accounts for interlayer inorganic/organic anions such as Cl^−^, NO_3_^−^ or dodecylsulfate (DS^−^).^8^ LLHs have been recently postulated as excellent anion exchangers, precursors to unique functional oxides or optical phosphors.^9^ With respect to their magnetic properties – despite their potential interest – the list of examples is very scarce and is almost limited to the study of a series of yttrium and dysprosium derivatives, showing a rich phenomenology including Single Ion Magnetic behaviour.^10,11^

However, the use of LLHs as low-temperature magnetic coolers has not yet been examined. In this sense, gadolinium-based molecular materials have been postulated as excellent alternatives to the well-established magnetic refrigerants at liquid-helium temperatures,^12^ as they exhibit an enhanced magnetocaloric effect (MCE), *i.e.* a change in the magnetic entropy (Δ*S*_m_) and related adiabatic temperature following a change in the applied magnetic field (Δ*B*).^13,14^ In order to maximize the MCE, weak superexchange interactions in materials having high density of magnetic centres are highly desired; along this front the use of geometric spin frustration, like 2D triangular AF lattices, gives rise to regions of high density of states.^15^ Thus, the highly dense, almost hexagonal, 2D lattice of lanthanide cations bridged by hydroxide ligands present in LLHs, together with its low diamagnetic content, is a promising alternative to the list of extended materials with short bridges reported so far with MCE, which includes, among others, formates,^16,17^ phosphates^18^ and carbonates,^19^ being [Gd(HCOO)(OAc)_2_(H_2_O)_2_],^20^ [Gd(C_4_O_4_)(OH)(H_2_O)_4_]_*n*_,^21^ [Gd(C_2_O_4_)(H_2_O)_3_Cl],^22^ and [Gd(cit)(H_2_O)],^23^ the only 2D structures. Importantly, the ability of LLHs to be delaminated^3^ provides additional advantages for rapid heat dissipation, as the delaminated material could be placed on a substrate thus enhancing heat transportation.^24,25^ Herein, we report the MCE of two Gd^3+^-based layered hydroxides, namely pristine Gd_2_(OH)_5_Cl·1.5H_2_O (LLH-1), and the Gd hydroxide intercalated with dodecyl sulfate Gd_2_(OH)_5_DS·*n*H_2_O (LLH-2, DS^−^ = C_12_H_25_SO_3_^−^). Their large MCE is quantitatively superior to almost all the best Gd-based clusters, and is similar to that recently reported for extended frameworks. Given the excellent processability exhibited by these compounds and their large-scale production, the Gd-LLHs represent an avenue worth being explored.

The synthesis of LLH-1 has been developed following the homogeneous alkalization route using NaCl as the anion source (see ESI† for additional experimental details and further characterization).^8^ The intercalation of DS^−^ was performed using the approach reported by Hu and co-workers.^26^Fig. 1 shows the crystalline structure of the layers,^27^ which contains three crystallographic distinct sites for gadolinium with two different environments, one 8-fold coordinated unit in a dodecahedron environment, [Gd(OH)_7_(H_2_O)], and two 9-fold coordinated units with a monocapped square antiprism geometry, [Gd(OH)_8_(H_2_O)].

**Fig. 1:**
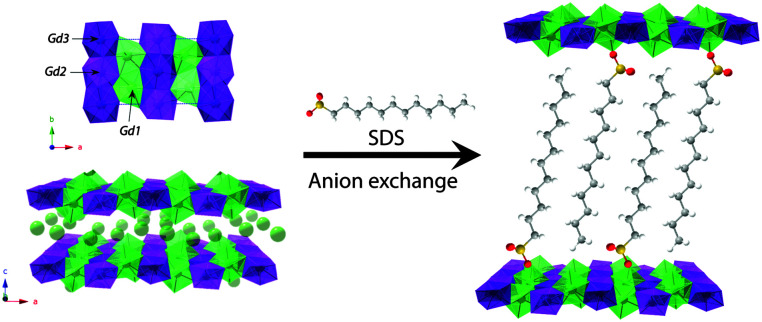
Gd_2_(OH)_5_Cl·1.5H_2_O (LLH-1) crystal structure viewed along the *c*-axis (top) and *b*-axis (bottom). Schematic representation of the dodecyl sulphate intercalation (C_12_H_25_SO_3_^−^) giving rise to LLH-2 exhibiting an expanded interlayer space. The 8-coordinated dodecahedra and 9-coordinated monocapped square antiprisms are highlighted in green and purple colours, respectively.

The crystal structure is composed of edge-sharing polyhedra, with the hydroxyl groups acting as *μ*_3_-bridges between the Gd centers yielding very short Gd⋯Gd distances (3.957, 3.944 and 3.662 Å). The powder X-ray diffraction (PXRD) pattern of LLH-1 shows sharp and intense peaks denoting high crystallinity (Fig. S4, ESI†). In this case, the sheets are separated by Cl^−^ anions, giving an interlayer distance of *ca.* 8.4 Å. The PXRD pattern of LLH-2 presents a shift of the diffraction peaks towards lower 2*θ* values resulting in an increased interlayer space of *ca.* 2.5 nm, indicative of the interdigitated disposition of the DS^−^ molecules within the interlamellar space.

The morphology of the as-synthesized materials has been studied by scanning electron microscopy, revealing a homogeneous distribution of anisotropic platelet-like particles less than one micron in lateral dimensions before and after the intercalation process (Fig. S5 and S6, ESI†). Exfoliation of LLH-2 produces nanosheets of *ca.* 3 nm thickness, as estimated by AFM (Fig. S7, ESI†), which correspond to mono- or bi-layers, as previously described.^26,28^

Fig. 2 depicts the variable temperature magnetic susceptibility measurements above 2 K of both samples in an applied dc field of 0.1 T. At room temperature, the *χ*_m_*T* value is 15.7 emu mol^−1^ K, which is in good agreement with the spin-only value expected for two uncoupled Gd^3+^ centres (2 × 7.875 emu mol^−1^ K). Upon cooling below 100 K, *χ*_m_*T* decreases significantly, denoting dominant antiferromagnetic interactions, likely extending over the *ab* planes. No clear differences could be observed between the compact LLH-1 and the expanded LLH-2 system, except for the lowest temperatures, *T* < 3 K (see Fig. 2). As shown by the solid line, the data can be fitted between 3 K and 300 K by a Curie–Weiss law *χ*_m_ = *C*/(*T* – *θ*_CW_), where *C* = *g*^2^*μ*_B_^2^*s*(*s* + 1)/(3*k*_B_), for *s* = 7/2, *g* = 2.0 and *θ*_CW_ = 3.1 K. Using the mean-field expression for the Curie–Weiss temperature *θ*_CW_ = 2*z*|*J*|*s*(*s* + 1)/(3*k*_B_), where *z* is the number of nearest neighbours, we find the estimate *zJ*/*k*_B_ ≈ −0.3 K for the antiferromagnetic interaction strength within the *ab* planes, for both compounds. Magnetization (*M*) *versus* applied field data, collected for the 2 K < *T* < 10 K range and applied fields up to 5 T, corroborate the paramagnetic susceptibility of both compounds (Fig. S8, ESI†).

**Fig. 2:**
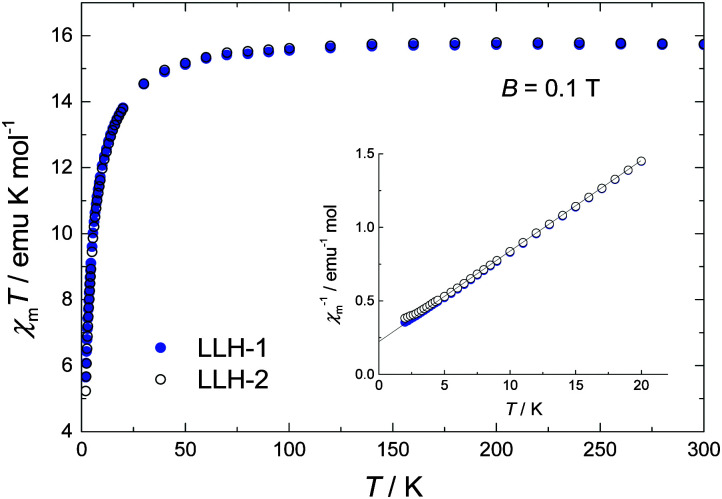
Temperature-dependencies of the magnetic susceptibility product, *χ*_m_*T* at 2–300 K with a dc field of 0.1 T for LLH-1 and LLH-2, denoting predominant antiferromagnetic interactions. Inset: the same set of data as *χ*_m_^−1^*vs. T* for *T* < 20 K, together with the fit to the Curie–Weiss law (solid line).

Specific heat (*c*) measurements down to *ca.* 0.3 K reveal the differences between the two compounds (Fig. 3 and 4). At higher temperatures, *c* is dominated by a nonmagnetic contribution arising from thermal vibrations of the lattice, which can be modelled by the Debye–Einstein model.^29^ The lattice specific heat simplifies to a *c*/*R* = *aT*^3^ dependence at the lowest temperatures, where *R* is the gas constant and *a* = 1.5 × 10^−4^ K^−3^ and 1.0 × 10^−3^ K^−3^ for LLH-1 and LLH-2, respectively. This difference indicates a stiffer structure for LLH-1, as indeed expected.

**Fig. 3:**
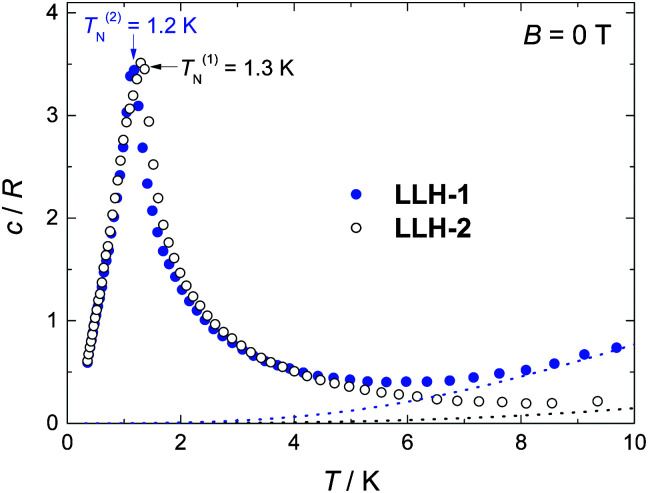
Temperature dependencies of the zero-applied-field specific heat, for LLH-1 and LLH-2, normalized to the gas constant (*R*). The peaks at *T*^(1)^_N_ = 1.3 K and *T*^(2)^_N_ = 1.2 K denote the transition to a long-range magnetically ordered state for LLH-1 and LLH-2, respectively. The dotted lines represent the Debye–Einstein model for the lattice specific heat.

**Fig. 4:**
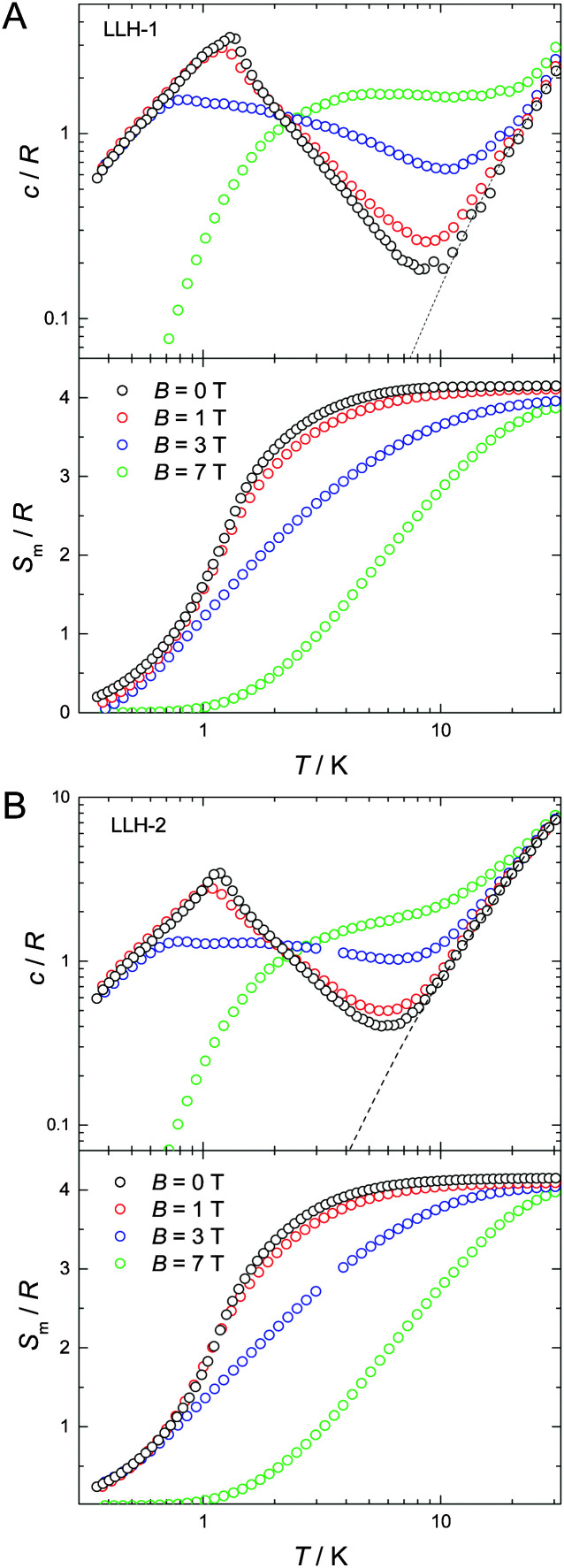
Temperature dependencies of the molar specific heat and magnetic entropy for LLH-1 (A) and LLH-2 (B). Top: Temperature-dependencies of the specific heat, *c*, normalized to the gas constant (*R*) and measured for several applied magnetic fields, as labelled. The dotted line represents the Debye–Einstein model for the lattice specific heat. Bottom: Corresponding temperature-dependencies of the magnetic entropy, *S*_m_, as obtained from the magnetic contribution to the specific heat. *S*_m_ saturates to the maximum entropy value per mole involved corresponding to two Gd^3+^ ions, *i.e.*, 2 × *R* ln(2*s*_Gd_ + 1) ≈ 4.2*R*.

At low temperatures and large applied fields, the magnetic contribution to the specific heat (*c*_m_), as resulting from subtracting the lattice contribution to the total specific heat, exhibits the typical Schottky-type anomalies originated by the splitting of the *s* = 7/2 multiplet (Fig. 3). For a zero-applied field, the specific heat is characterized by a lambda-like peak denoting the occurrence of magnetic phase transition at *T*^(1)^_N_ = 1.3 K for LLH-1 and a slightly lower *T*^(2)^_N_ = 1.2 K for LLH-2 (Fig. 4). Since no phase transition can occur at nonzero temperature for isotropic 2D magnetic lattices, the observed *T*_N_s could either be ascribed to an Ising-type anisotropy or 3D coupling.^30^ We disregard the latter option since the structure of the compounds should favour stronger intralayer magnetic fluctuations that give rise to a broad bump in *c* for temperatures higher than that of the phase transition, induced by the weaker interlayer coupling, in clear disagreement with the measurements (Fig. 4). Therefore, one has to conclude that anisotropy crossover from Heisenberg to Ising takes place in the critical temperature region. We argue that this behaviour can be understood in terms of dipolar anisotropy. Let us first hypothetically assume that no superexchange interactions are present. If so, then the dipolar energy is minimized when all spins align ferromagnetically on the *ab* planes.^31^ Let us next add intralayer antiferromagnetic superexchange interactions, significantly stronger than the dipolar ones. If so, then aligning the antiferromagnetically-coupled spins on the *ab* planes would no longer be energetically favourable. In this case, neglecting any source of geometric spin frustration, spins would preferably point perpendicular to the layers, *i.e.*, along the *c* axis. Using the structure of LLH-1 and associating each Gd^3+^ spin to a point-dipole with *s* = 7/2, we have calculated the dipolar energies (*E*_d_) for both the aforementioned magnetic structures. For LLH-1, we obtain *E*^(F//*ab*)^_d_ = −0.7 K and *E*^(AF//*c*)^_d_ = −0.2 K, respectively. For LLH-2, we repeat the calculation using the same structure except for the distance between the layers, which we increase up to the experimentally determined value of 2.5 nm. We thus obtain significantly weaker energies, *i.e.*, *E*^(F//*ab*)^_d_ = −0.4 K and *E*^(AF//*c*)^_d_ = −0.1 K, respectively. Next, from the difference in dipolar energy for the two orientations considered, we obtain the anisotropy field *B*_a_ = 0.10 T and 0.06 T for LLH-1 and LLH-2, respectively. Note that we likely overestimate these values since full collinear magnetic ordering is hindered by the geometric spin frustration on the *ab* planes. The so-obtained *B*_a_ values are significantly smaller than the exchange field *B*_ex_ = 2*z*|*J*|*s*/(*gμ*_B_) ≈ 1.6 T, thus yielding (*B*_a_/*B*_ex_) ≈ 6 × 10^−2^ and 4 × 10^−2^ for LLH-1 and LLH-2, respectively. A comparable (*B*_a_/*B*_ex_) ≈ 8 × 10^−2^ is found in the 2D antiferromagnet GdBa_2_Cu_3_O_6+*x*_, whose magnetic ordering mechanism closely resembles the one reported here.^32^ As for GdBa_2_Cu_3_O_6+*x*_ and other 2D antiferromagnets,^29,31^ the ordering temperature depends very weakly on (*B*_a_/*B*_ex_),^33^ in agreement with the measured *T*_N_s for LLH-1 and LLH-2.

Finally, we evaluate the MCE for both compounds by determining the magnetic entropy change, −Δ*S*_m_, as a function of temperature and for selected applied field changes, Δ*B*, following well-known data-processing procedures.^13^ From the magnetic entropy data in Fig. 4, we straightforwardly obtain the −Δ*S*_m_(*T*,Δ*B*) curves depicted in Fig. 5. As can be seen, the curves for Δ*B* = 1 T and 3 T agree nicely with the ones calculated by applying the Maxwell relation 
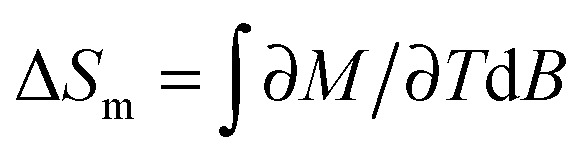
 to the magnetization data in Fig. S8 (ESI†), thus validating the two independent derivations employed. Note that, not unexpectedly, the −Δ*S*_m_(*T*,Δ*B*) curves for both compounds overlap one another for the same set of *T* and Δ*B* values (Fig. 5). Clearly, the intralayer superexchange interaction (which has the same strength for both compounds) is mainly responsible for the MCE data shown in Fig. 5, since the weak dipolar interactions become significant only at the lowest *B* and *T*, *i.e.*, well below the temperatures that correspond to the maxima of −Δ*S*_m_(*T*,Δ*B*). For Δ*B* = 1 T, the MCE is rather small and clearly hindered by the intralayer antiferromagnetic interactions. Higher fields promote larger magnetic decoupling and the field dependence of −Δ*S*_m_ increases notably (Fig. 5). For the largest field change Δ*B* = 7 T, −Δ*S*_m_ reaches 3.0*R* at *T* = 2.2 K, which is smaller than the maximum entropy value per mole involved, *i.e.*, 2 × *R* ln(2*s* + 1) ≈ 4.2*R*. Notwithstanding Δ*B* = 7 T is not yet sufficient for achieving the full magnetocaloric potential, the measured maximum entropy change is significantly large, as readily evident by expressing −Δ*S*_m_ in the most common choice of units, *i.e.*, −Δ*S*_m_ = 51.9 and 42.0 J kg^−1^ K^−1^ at *T* = 2.2 K and Δ*B* = 7 T for LLH-1 and LLH-2, respectively. This results from the high magnetic/nonmagnetic ratio (relatively low molecular mass) of both compounds. LLH-1 neatly surpasses the values reported for 2D complexes so far,^20–23^ and compares favourably with the recently reported records.^17,18^

**Fig. 5:**
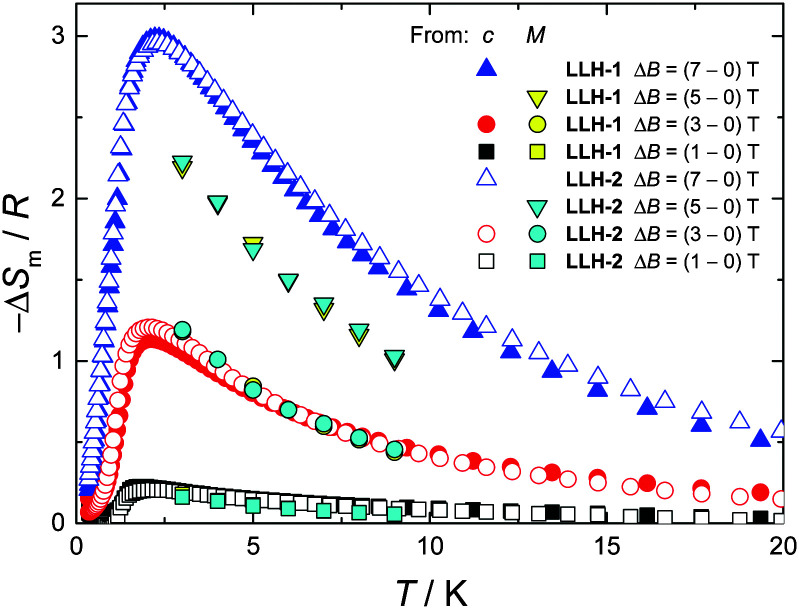
Temperature dependencies of the magnetic entropy change, −Δ*S*_m_, normalized to the gas constant *R*, obtained from magnetization (*M*) and specific heat (*c*) data, for the applied-field changes Δ*B* and compounds LLH-1 and LLH-2, as labelled.

In summary, we report for the very first time the cryogenic MCE of a layered lanthanide hydroxide, exhibiting significantly large values for the ≈(1.5–7) K temperature range. The interlayer dipolar interactions have been evaluated by hybridization of the pristine material with DS^−^ molecules. Magnetic ordering of the Gd^3+^ ions develops in the form of a 2D Heisenberg–Ising crossover behaviour near the Néel temperature, as induced by the dipolar interactions. The intrinsic 2D spin frustrated topology could be considered as an extension of the 0D heptametallic gadolinium molecule,^15^ serving as an ideal example of extended triangular AF nets where dipolar and exchange contributions compete. Furthermore, as these layered materials can be exfoliated into unilamellar nanosheets,^3^ produced in a large scale,^8^ and processed into complex architectures,^34^ they can be proposed as active elements for on-chip magnetic microrefrigerators^25^ and excellent alternatives to the existing commercial low-temperature refrigerants.

We are grateful to the EU (ERC Advanced Grant SPINMOL), the Spanish MINECO (Projects MAT-2014-56143-R, CTQ-2014-59209-P and FEDER-MAT2012-38318-C03-01), and the Generalitat Valenciana (Prometeo Program and ISIC-Nano). Support from INNCIDE program through Vicerectorat d'lnvestigació i Política Científica of the University of Valencia is also acknowledged. G. A. thanks the EU for a Marie Curie Fellowship (FP7/2013-IEF-627386). G.M.E. thanks the Spanish MINECO for a Ramón y Cajal Fellowship.

## Supplementary Material

CC-051-C5CC05150A-s001

## References

[cit1] Ferrari A. C., Bonaccorso F., Fal’ko V., Novoselov K. S., Roche S., Bøggild P., Borini S., Koppens F. H. L., Palermo V., Pugno N., Garrido J. A., Sordan R., Bianco A., Ballerini L., Prato M., Lidorikis E., Kivioja J., Marinelli C., Ryhänen T., Morpurgo A., Coleman J. N., Nicolosi V., Colombo L., Fert A., Garcia-Hernandez M., Bachtold A., Schneider G. F., Guinea F., Dekker C., Barbone M., Sun Z., Galiotis C., Grigorenko A. N., Konstantatos G., Kis A., Katsnelson M., Vandersypen L., Loiseau A., Morandi V., Neumaier D., Treossi E., Pellegrini V., Polini M., Tredicucci A., Williams G. M., Hong B. H., Ahn J.-H., Kim J. M., Zirath H., van Wees B. J., van der Zant H., Occhipinti L., Matteo A. D., Kinloch I. A., Seyller T., Quesnel E., Feng X., Teo K., Rupesinghe N., Hakonen P., Neil S. R. T., Tannock Q., Löfwander T., Kinaret J. (2015). Nanoscale.

[cit2] Wang Q., O’Hare D. (2012). Chem. Rev..

[cit3] Ma R., Sasaki T. (2015). Acc. Chem. Res..

[cit4] Abellán G., Martí-Gastaldo C., Ribera A., Coronado E. (2015). Acc. Chem. Res..

[cit5] Abellán G., Coronado E., Martí-Gastaldo C., Ribera A., Jordá J. L., García H. (2014). Adv. Mater..

[cit6] Abellán G., Jordá J. L., Atienzar P., Varela M., Jaafar M., Gómez-Herrero J., Zamora F., Ribera A., García H., Coronado E. (2015). Chem. Sci..

[cit7] Ma R., Sasaki T. (2010). Adv. Mater..

[cit8] Geng F., Matsushita Y., Ma R., Xin H., Tanaka M., Izumi F., Iyi N., Sasaki T. (2008). J. Am. Chem. Soc..

[cit9] McIntyre L. J., Jackson L. K., Fogg A. M. (2008). Chem. Mater..

[cit10] Monteiro B., Pereira C. C. L., Coutinho J. T., Pereira L. C. J., Marçalo J., Almeida M. (2013). Eur. J. Inorg. Chem..

[cit11] Monteiro B., Coutinho J. T., Pereira C. C. L., Pereira L. C. J., Marçalo J., Almeida M., Baldoví J. J., Coronado E., Gaita-Ariño A. (2015). Inorg. Chem..

[cit12] Sharples J. W., Collison D. (2013). Polyhedron.

[cit13] Evangelisti M., Brechin E. K. (2010). Dalton Trans..

[cit14] Liu J.-L., Chen Y.-C., Guo F.-S., Tong M.-L. (2014). Coord. Chem. Rev..

[cit15] Sharples J. W., Collison D., McInnes E. J. L., Schnack J., Palacios E., Evangelisti M. (2014). Nat. Commun..

[cit16] Sibille R., Mazet T., Malaman B., François M. (2012). Chem. – Eur. J..

[cit17] Lorusso G., Sharples J. W., Palacios E., Roubeau O., Brechin E. K., Sessoli R., Rossin A., Tuna F., McInnes E. J. L., Collison D., Evangelisti M. (2013). Adv. Mater..

[cit18] Palacios E., Rodríguez-Velamazán J. A., Evangelisti M., McIntyre G. J., Lorusso G., Visser D., de Jongh L. J., Boatner L. A. (2014). Phys. Rev. B: Condens. Matter Mater. Phys..

[cit19] Chen Y.-C., Qin L., Meng Z.-S., Yang D.-F., Wu C., Fu Z., Zheng Y.-Z., Liu J.-L., Tarasenko R., Orendáč M., Prokleška J., Sechovský V., Tong M.-L. (2014). J. Mater. Chem. A.

[cit20] Lorusso G., Palacios M. A., Nichol G. S., Brechin E. K., Roubeau O., Evangelisti M. (2012). Chem. Commun..

[cit21] Biswas S., Adhikary A., Goswami S., Konar S. (2013). Dalton Trans..

[cit22] Meng Y., Chen Y.-C., Zhang Z.-M., Lin Z.-J., Tong M.-L. (2014). Inorg. Chem..

[cit23] Liu S.-J., Xie C.-C., Jia J.-M., Zhao J.-P., Han S.-D., Cui Y., Li Y., Bu X.-H. (2014). Chem. – Asian J..

[cit24] Corradini V., Ghirri A., Candini A., Biagi R., del Pennino U., Dotti G., Otero E., Choueikani F., Blagg R. J., McInnes E. J. L., Affronte M. (2013). Adv. Mater..

[cit25] Lorusso G., Jenkins M., González-Monje P., Arauzo A., Sesé J., Ruiz-Molina D., Roubeau O., Evangelisti M. (2013). Adv. Mater..

[cit26] Hu L., Ma R., Ozawa T. C., Sasaki T. (2010). Chem. – Asian J..

[cit27] Geng F., Ma R., Sasaki T. (2010). Acc. Chem. Res..

[cit28] Lee B.-I., Lee K. S., Lee J. H., Lee I. S., Byeon S.-H. (2009). Dalton Trans..

[cit29] Evangelisti M., Luis F., de Jongh L. J., Affronte M. (2006). J. Mater. Chem..

[cit30] Jongh L. J. D., Miedema A. R. (2001). Adv. Phys..

[cit31] de Jongh L. J. , Magnetic Properties of Layered Transition Metal Compounds, Springer Science & Business Media, 1990

[cit32] Nehrke K., Pieper M. W. (1995). Phys. Rev. B: Condens. Matter Mater. Phys..

[cit33] Binder K., Landau D. P. (1976). Phys. Rev. B: Solid State.

[cit34] Lee B.-I., Lee E., Byeon S.-H. (2012). Adv. Funct. Mater..

