# Supplementary material for: Layered gadolinium hydroxides for low-temperature magnetic cooling
Source: Chem Commun (Camb). 2015 Jul 29;51(75):14207–10. doi: 10.1039/c5cc05150a (PMC4720933; doi:10.1039/c5cc05150a)
Supplement: CC-051-C5CC05150A-s001 [file CC-051-C5CC05150A-s001.pdf]

Electronic Supplementary Material (ESI) for ChemComm.
This journal is © The Royal Society of Chemistry 2015

Supplementary Information

Layered Gadolinium Hydroxides for Low-Temperature Magnetic Cooling

Gonzalo Abellán,[a§†] Guillermo Mínguez Espallargas,[a§] Giulia Lorusso,[b] Marco Evangelisti,*[b] and Eugenio Coronado*[a]

[a] *Instituto de Ciencia Molecular (ICMol), Universidad de Valencia, 46980, Valencia, Spain.*

E-mail: eugenio.coronado@uv.es

[b] *Instituto de Ciencia de Materiales de Aragón (ICMA) and Departamento de Física de la Materia Condensada, CSIC–Universidad de Zaragoza, Pedro Cerbuna 12, 50009 Zaragoza, Spain.*

Email: evange@unizar.es

§ These authors contributed equally to this work.

† Current address: Department of Chemistry and Pharmacy and Institute of Advanced Materials and Processes (ZMP), University Erlangen-Nürnberg, Henkestr. 42, 91054 Erlangen and Dr.-Mack Str. 81, 90762 Fürth, Germany.

Contents

SI 1. Experimental Information.

SI 2. FT-IR Spectra of LLH-2.

SI 3. Thermogravimetric (TGA/DTA) Analysis of LLH-1 and LLH-2.

SI 4. X-ray powder diffraction.

SI 5. FESEM analysis of LLH-1 and LLH-2.

SI 6. Particle size analysis.

SI 7. AFM analysis.

SI 8. Isothermal magnetization curves for LLH-1 and LLH-2.

References

SI 1. Experimental Information.

The starting materials $GdCl_3 \cdot 6H_2O$ (Aldrich), NaCl (Panreac), hexamethylenetetramine (HMT) (Aldrich), and $NaC_{12}H_{25}SO_3$ (Aldrich) were purchased from commercial sources and used as received.

Synthesis of LLH-1. $Gd_2(OH)_5Cl \cdot 1.5H_2O$ was synthesized according to the homogeneous alkilization route by using NaCl as the anion source and HMT as the ARR.[1] A mixture of $GdCl_3 \cdot 6H_2O$ (0.659 g; 5 mmol), NaCl (0.950 g; 32.5 mmol), and HMT (0.175 g; 2.5 mmol) was dissolved in 500 cm^3 of decarbonated Milli-Q water, and the solution was heated at refluxing temperature under continuous magnetic stirring (900 rpm) and argon gas protection. After 10 hours, the resulting finely divided white powder was filtered, washed thoroughly with Milli-Q water and ethanol, and dried at room temperature under vacuum.

Synthesis of LLH-2. LLH-2 was obtained by chloride-exchange of the as-synthesized $Gd_2(OH)_5Cl \cdot 1.5H_2O$ in the presence of an excess of dodecyl sulfate anions.[2] In a typical procedure, 100 mg of **LLH-1** were suspended in 100 mL of Milli-Q water containing 5 mmol of $C_{12}H_{25}SO_3^-$ and stirred 20 h at room temperature. The product was recovered by filtration, washed thoroughly with Milli-Q water and ethanol, and dried at room temperature under vacuum.

Exfoliation of LLH-2. **LLH-2** powder (0.010 g) was dispersed in 50 mL of formamide. The mixture was vigorously agitated by orbital shaking (*ca*. 200 cycles per min) for 3 days. The nanosheet suspension was obtained after separating an unexfoliated component by centrifugation at 12 000 rpm for 90 min. Atomic Force Microscopy (AFM) measurements were collected in a Multimode atomic force microscope (Veeco Instruments, Inc.). Typically, a freshly diluted nanosheet suspension was deposited onto a clean Si wafer by spin coating at 6000 rpm. The images were obtained with a Si tip (frequency and *K* of *ca*. 300 kHz and 42

N·m^{-1}, respectively) using the tapping-mode in air at room temperature. Images were recorded with a 0.5–1 Hz scan rate. Processing and analysis of the images were carried out using the Nanotec WSXM-4.0 Beta 6.5 sofware (http://www.nanotec.es).[3]

Physical Characterization

Field emission scanning electron microscopy (FESEM) studies were performed on a Hitachi S-4800 microscope operating at an accelerating voltage of 20 kV and without metallization of the samples.

Thermogravimetric analysis (TGA) of all compounds were carried out with a Mettler Toledo TGA/SDTA 851 apparatus in the 25–800 ºC temperature range at a 10 ºC min^{-1} scan rate and an air flow of 30 mL·min^{-1}.

X-ray diffraction (XRD) patterns were obtained using a Philips X'Pert diffractometer using the copper radiation (Cu-Kα = 1.54178 Å).

Infrared spectra were recorded in a FT-IR Nicolet 5700 spectrometer in the 4000–400 cm^{-1} range with a nominal resolution of 0.4 cm^{-1}.

Magnetic measurements were performed using a Quantum Design MPMS-XL SQUID magnetometer. The measured values were corrected for the experimentally measured contribution of the sample holder, while the derived susceptibilities were corrected for the diamagnetism of the samples, estimated from Pascal's tables.

Specific heat measurements were carried out at temperatures down to ca. 0.3 K by using a Quantum Design 9T-PPMS, equipped with a ^{3}He cryostat. The experiments were performed on thin pressed pellets (ca. 1 mg) of a polycrystalline sample, thermalised by ca. 0.2 mg of Apiezon N grease, whose contribution was subtracted using a phenomenological expression.

Dipolar Calculations.

Extensive calculations of the dipolar interaction energy were performed for s = 7/2 point-like dipoles arranged in analogous crystallographic lattices to that of **LLH-1** and **LLH-2**, and using periodic boundary conditions.

SI 2. FT-IR Spectra of LLH-2.

The FT-IR spectra of **LLH-2** confirms the successful DS^- intercalation. Absorption broad band at *ca*. 3350 cm^{-1} and the peak at around 1637 cm^{-1} reveals the presence water hydration in the structure, and they are assignable to the O-H stretching vibrations and the H-O-H bending mode of water, respectively. The band observed in the range 3400-3700 cm^{-1} (centered at around 3500 cm^{-1}) is attributed to the presence of hydroxyl groups (OH^-). The intercalation of the dodecyl sulphate has been corroborated by the detection of its characteristic bands. The absorption bands at *ca*. 1060, 1200 and 1235 cm^{-1} are associated with the stretching modes of the sulphate (SO_3^-).[4] Moreover the strong absorption bands at 2920 and 2847 cm^{-1} are due to the asymmetric and symmetric CH_2 stretching vibrations, respectively. The weak band at *ca*. 2953 cm^{-1} is assigned to the stretching mode of the terminal CH_3 group of the hydrocarbon tail.

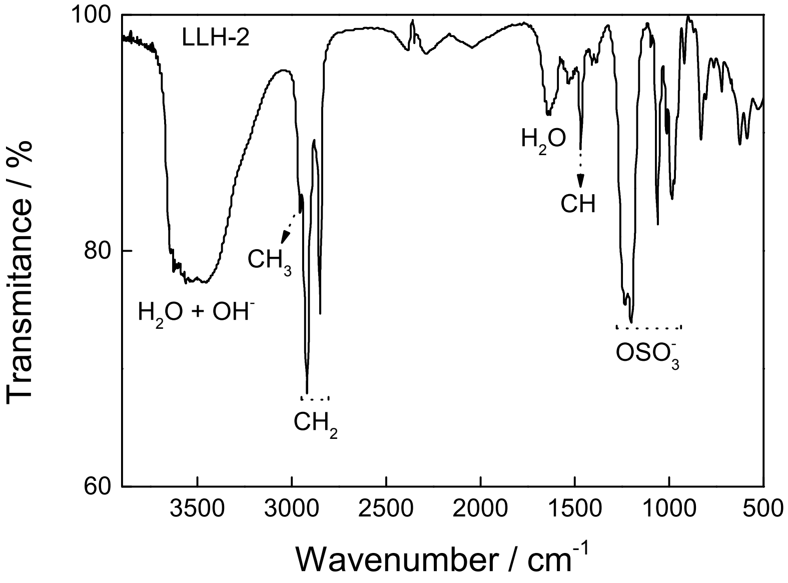

SI 3. Thermogravimetric (TGA/DTA) Analysis of LLH-1 and LLH-2. Thermal decomposition in air of **LLH-1** (top) and **LLH-2** (bottom) collected at a scan rate of 10 ºC·min^{-1}.

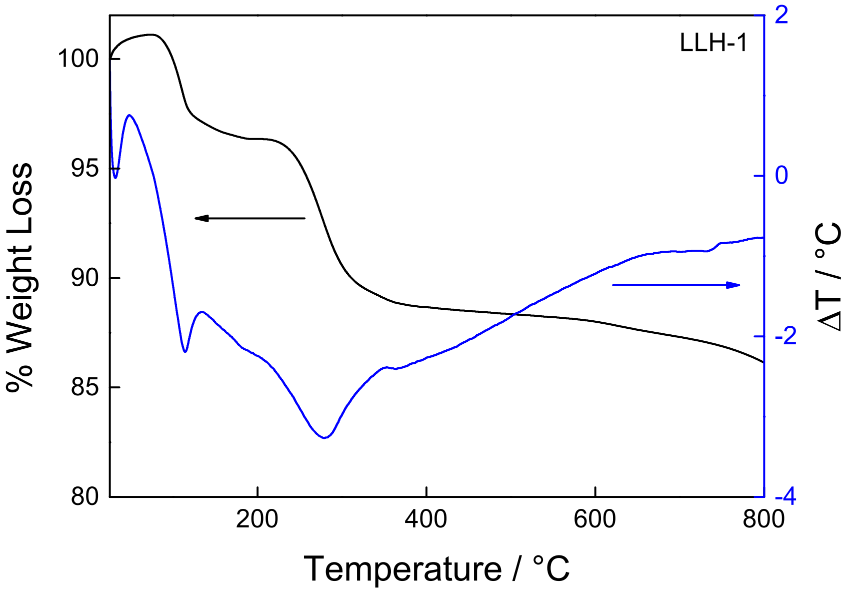

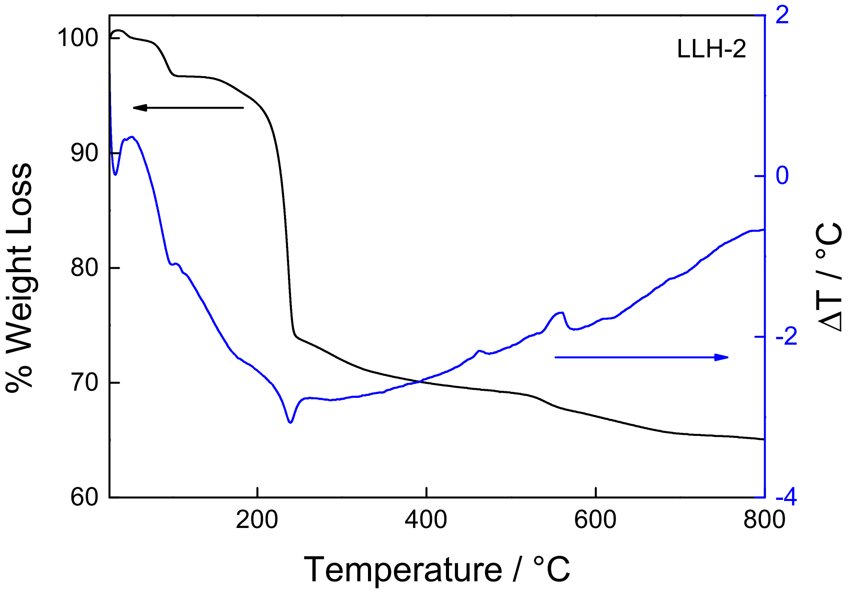

SI 4. X-ray Powder Diffraction. The powder X-ray diffraction (XRD) experiments were performed to verify the purity of the layered material **LLH-1** and the modification of the interlayer spacing due to the exchange of Cl^- to DS^- anions (*i.e.* formation of **LLH-2**).

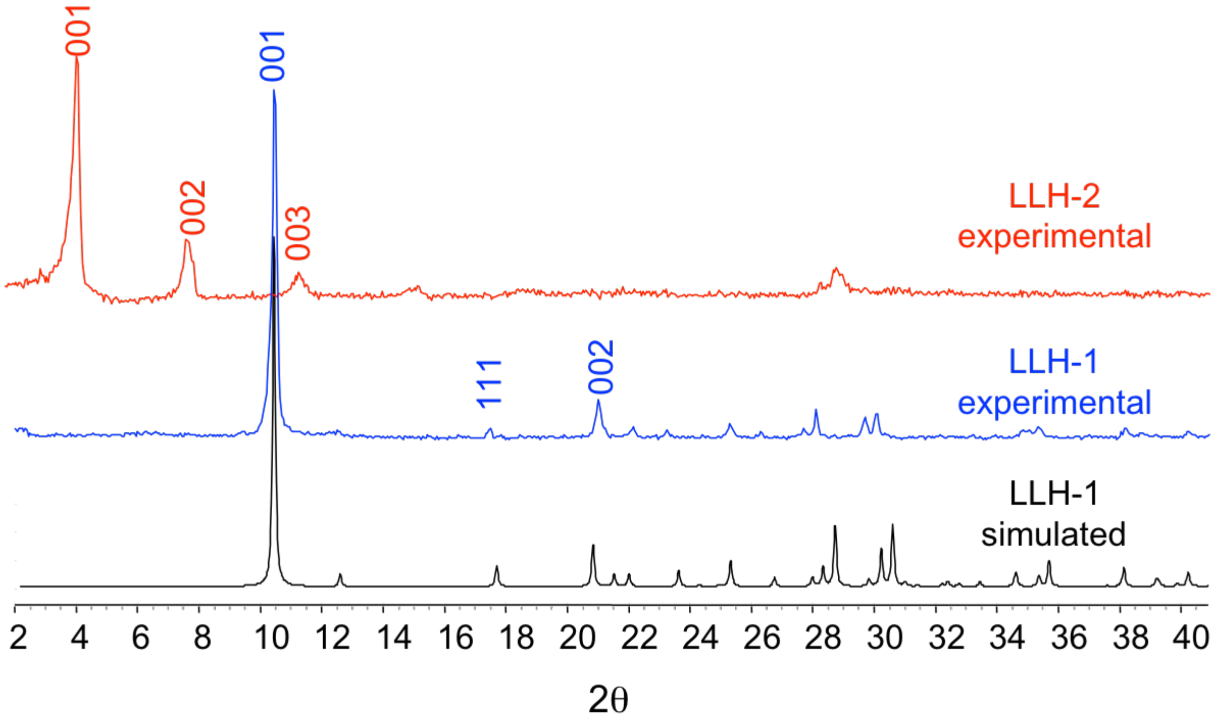

SI 5. FESEM Analysis of LLH-1 and LLH-2. (A and B) FESEM images of the **LLH-1** compound showing a highly homogeneous sample consisting on anisotropic platelet-like particles of sizes of several hundred of nanometres (*vide infra*). (C and D) FESEM images of **LLH-2** compound exhibiting the same homogeneity, in this case an increased thickness of the particles due to the anion exchange reaction is clearly observed.

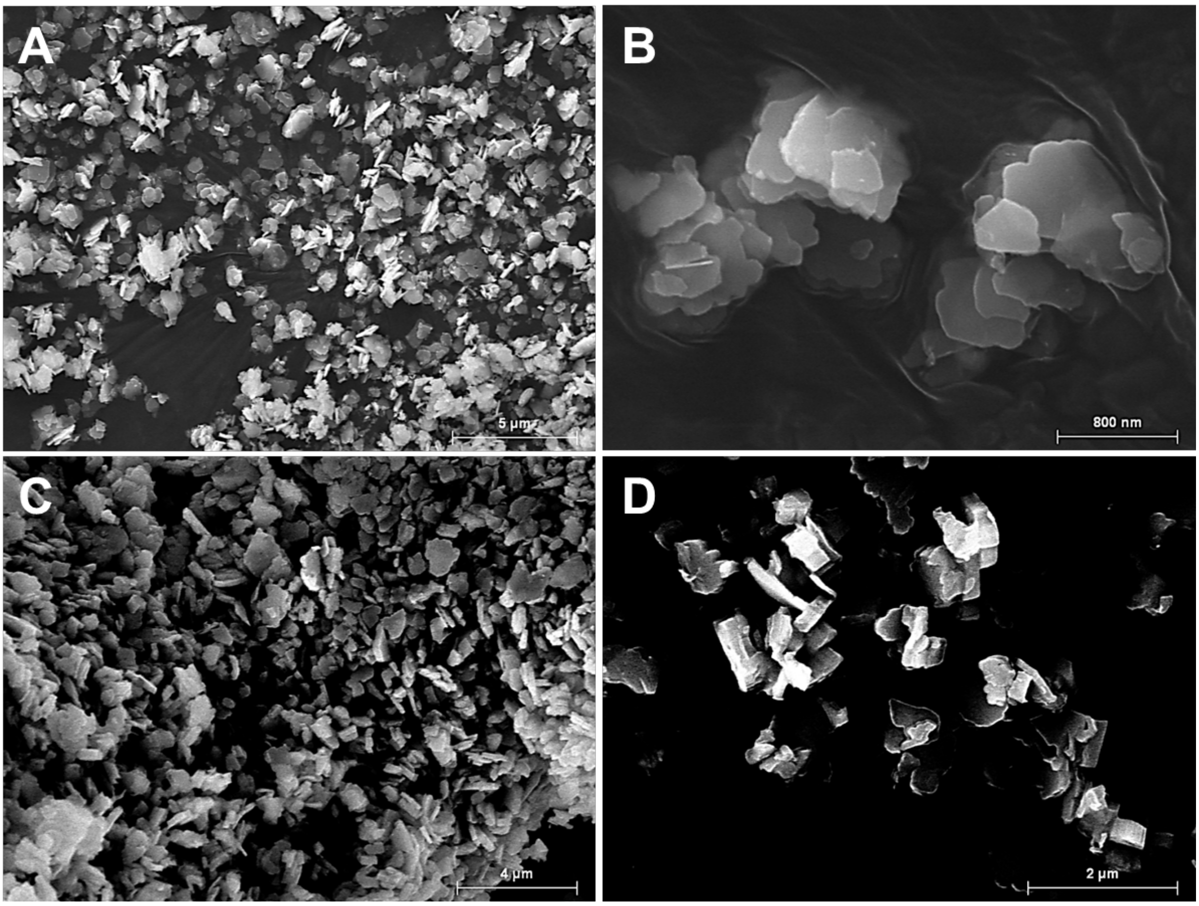

SI 6. Particle Size Analysis. Particle size distribution extracted from the FESEM analysis highlighting the average values obtained for the diameter and thickness of **LLH-1** (A and B) and **LLH-2** (C and D), respectively. Moreover, a precise correlation between the thickness of the platelets and their composition has been observed. In fact, while the lateral dimensions were almost retained, the thickness of the platelets increased by the anion exchange from Cl^- to DS^-. The ratio of the average particle thickness (from 69 to 229 nm; 3.3 times) before and after the exchange of Cl^- with the DS^- mixture was consistent with the ratio of the basal spacing (from 0.84 to 2.47 nm; 2.9 times) before and after the anion exchange determined by PXRD. These measurements confirm the topochemical reaction of the intercalation, as recently reported.[5,6]

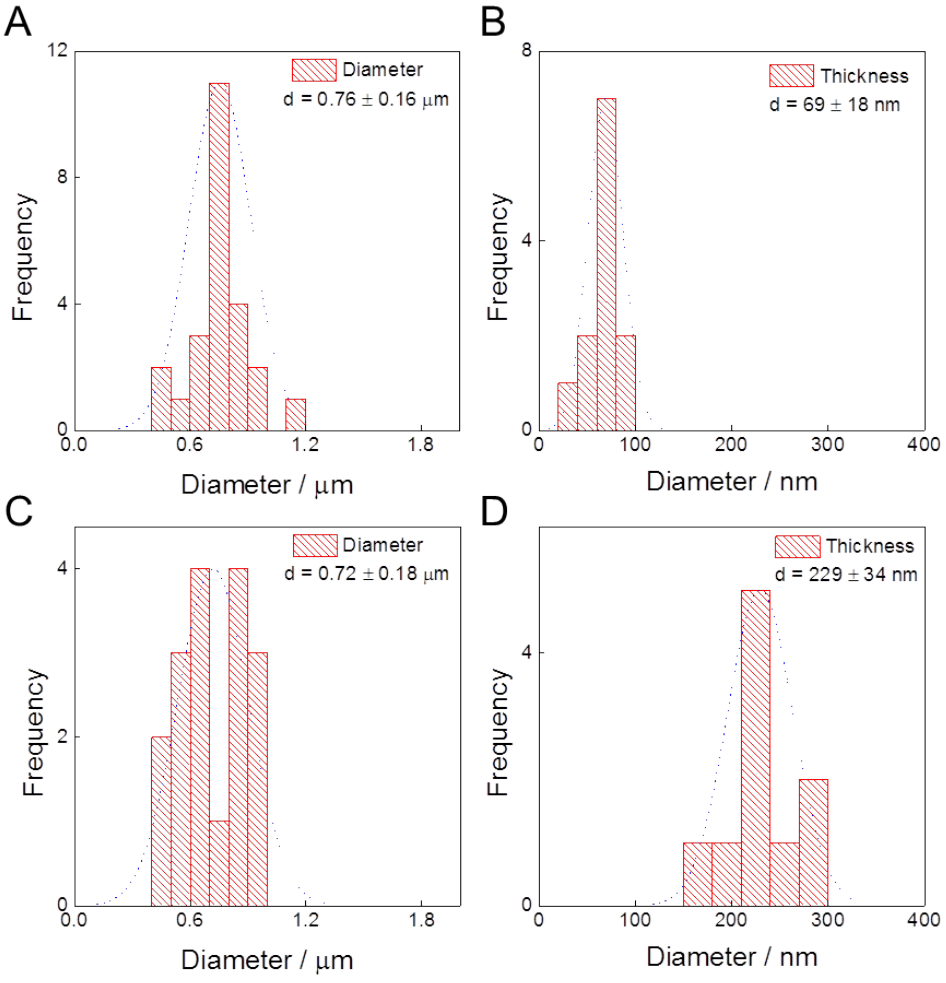

SI 6. AFM Analysis. (A) AFM topography image and the corresponding height profiles of the exfoliated Gadolinium hydroxide (LSH-2) deposited on a silicon wafer substrate by spin coating. (B) Magnification of the area highlighted in (A), showing a typical Gd hydroxide nanosheet.

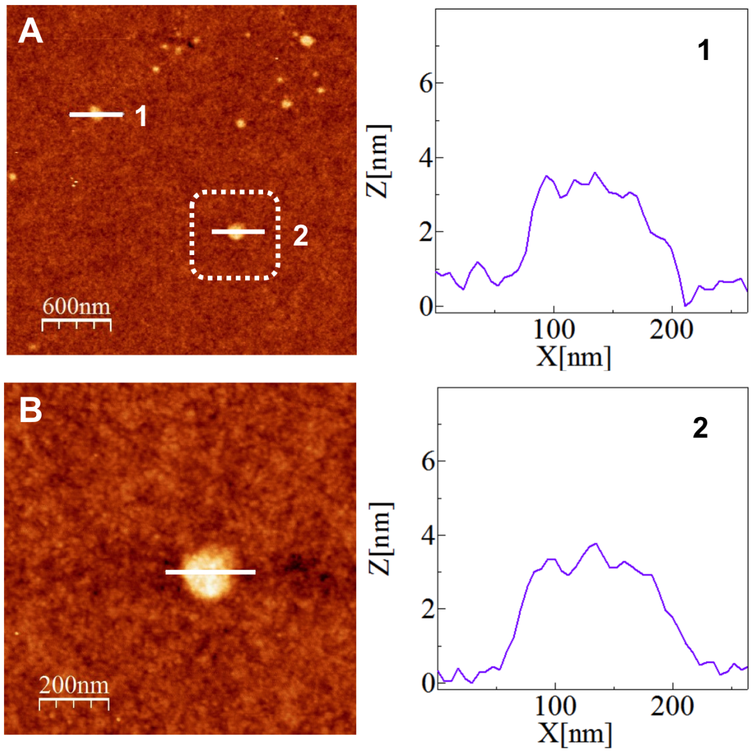

SI 8. Isothermal Magnetization Curves for LLH-1 and LLH-2. The temperature isothermal magnetization curves for **LLH-1** (top panel) and **LLH-2** (bottom panel), collected for temperatures ranging from 2 to 10 K.

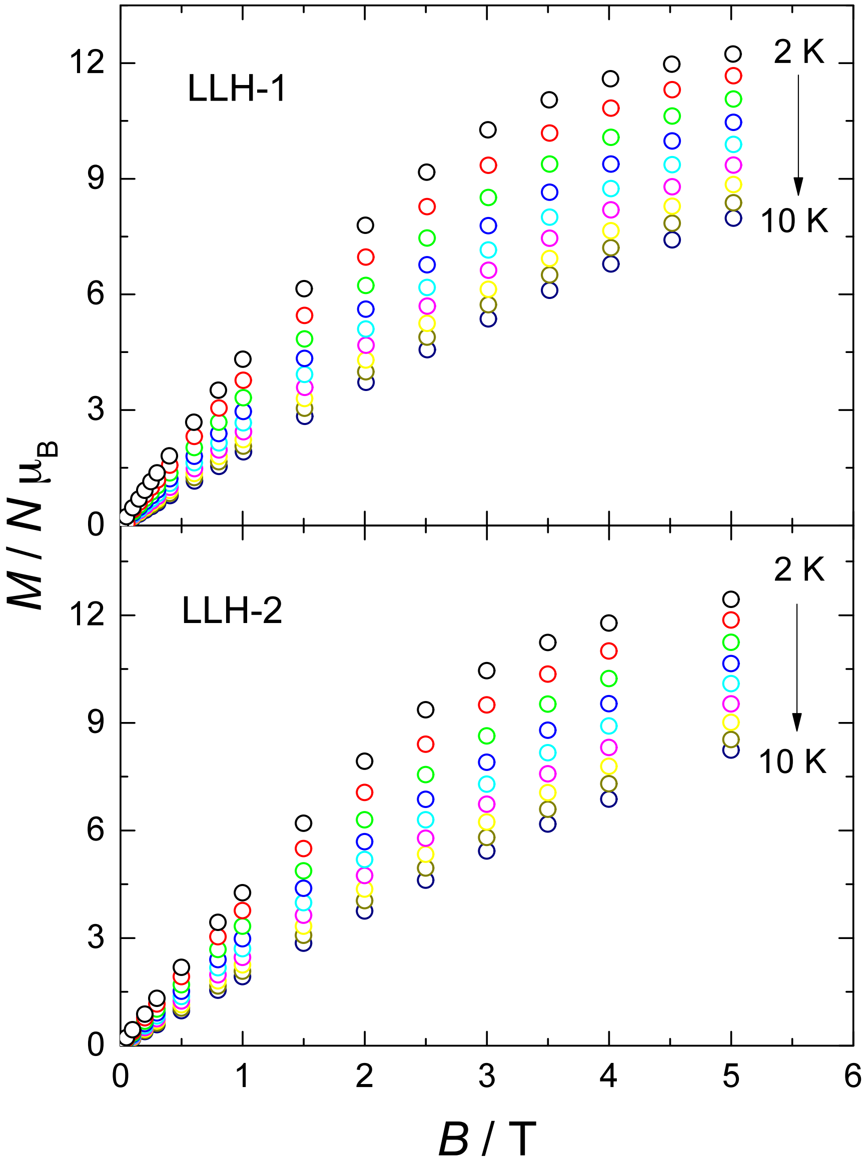

References

1 L. Hu, R. Ma, T. C. Ozawa and T. Sasaki, *Inorg. Chem.*, 2010, **49**, 2960–2968.

2 L. Hu, R. Ma, T. C. Ozawa and T. Sasaki, *Chem. – Asian J.*, 2010, **5**, 248–251.

3 I. Horcas, R. Fernández, J. M. Gómez-Rodríguez, J. Colchero, J. Gómez-Herrero and A. M. Baro, *Rev. Sci. Instrum.*, 2007, **78**, 013705.

4 B.-I. Lee, S.-Y. Lee and S.-H. Byeon, *J. Mater. Chem.*, 2011, **21**, 2916.

5 M. Ogawa and M. Hiramine, *Cryst. Growth Des.*, 2014, **14**, 1516–1519.

6 G. Abellán, J. L. Jordá, P. Atienzar, M. Varela, M. Jaafar, J. Gómez-Herrero, F. Zamora, A. Ribera, H. García and E. Coronado, *Chem Sci*, 2015, **6**, 1949–1958.
